# Relationship Between Resting Heart Rate and Microalbuminuria in Adults With Hypertension: National Health and Nutrition Examination Survey 2009–2018

**DOI:** 10.3389/fcvm.2022.739113

**Published:** 2022-04-12

**Authors:** Xiaodong Peng, Yukun Li, Xuesi Wang, Yanfei Ruan, Nian Liu

**Affiliations:** ^1^Department of Cardiology, Beijing Anzhen Hospital, Capital Medical University, Beijing, China; ^2^National Clinical Research Center for Cardiovascular Diseases, Beijing, China

**Keywords:** resting heart rate (RHR), hypertension, microalbuminuria (MAU), beta-blockers, early-stage renal diseases

## Abstract

**Background:**

The impact of elevated resting heart rate on early-stage renal dysfunction, manifesting as microalbuminuria, in hypertension is unclear. This study aimed to analyze the association between resting heart rate and microalbuminuria in patients with hypertension according to their blood pressure status. In addition, the effect of antihypertensive agents on this relationship was evaluated.

**Methods and Results:**

We searched the National Health and Nutrition Examination Survey for eligible participants from 2009 to 2018. Data on key parameters such as age, sex, blood pressure, heart rate, albumin creatinine ratio, and medication were collected for analysis. Subsequently, participants were classified according to the heart rate quartile and blood pressure status for subgroups assessment. A total of 5,692 participants were enrolled in this study. After adjusting the confounding factors, there was a linear association between resting heart rate and microalbuminuria in patients with hypertension (OR 1.184 [per 1 SD]; 95% CI: 1.101, 1.274; *P* < 0.001). However, the association between elevated resting heart rate and microalbuminuria was not significant in patients with uncontrolled hypertension (OR 1.092 [per 1 SD]; 95% CI: 0.935, 1.275; *P* = 0.269). The OR of the indirect effect of β-blockers on the risk of microalbuminuria incidence through heart rate was 0.926 (95% CI: 0.895, 0.956), while the direct effect was 1.374 (95% CI: 1.138, 1.662, *P* = 0.010). Similarly, dihydropyridine calcium channel blockers were associated with a higher prevalence of microalbuminuria (OR 1.300, 95% CI: 1.058, 1.597, *P* = 0.013), but the association between non-dihydropyridine calcium channel blockers and microalbuminuria was not significant (OR 1.207, 95% CI: 0.737, 1.978, *P* = 0.454).

**Conclusion:**

Elevated resting heart rate is associated with a high risk of microalbuminuria in untreated patients and patients with controlled hypertension. Although there is a linear association between heart rate and microalbuminuria, the use of β-blockers exhibits a significantly increase in the prevalence of microalbuminuria in hypertension. Likewise, dihydropyridine calcium channel blockers may increase the risk of microalbuminuria in hypertension.

## Introduction

Resting heart rate (RHR) is considered a practical marker of health and longevity. The impact of RHR on pathophysiological processes has been widely discussed from clinical and fundamental perspectives over decades. High RHR increases the risk of cardiovascular diseases, cancers, and all-cause mortality (1). Autonomic nervous system, a chronic disorder, causes an aberrant RHR, which contributes to vasoconstriction and hypertension concurrently (2). Previous studies have indicated that the RHR is tightly associated with progression of hypertension and prognosis of patients with hypertension, linking higher RHR with adverse outcome (3, 4). Further, experimental results have shown that elevated RHR exacerbates vascular oxidative stress and endothelial dysfunction and promotes atherogenesis (5). The detrimental effects of elevated RHR on arterial walls lead to the dysfunction of several organs, especially the heart and brain (6). Recently, the effect of RHR on the kidneys in individuals with hypertension has attracted increasing attention. In particular, the International Survey Evaluating Microalbuminuria Routinely by Cardiologists in Patients with Hypertension (I-SEARCH) has pointed out that high RHR is an independent risk factor for the development of microalbuminuria (MAU) in patients with hypertension (7).

MAU is a potent indicator for early-stage glomerular diseases and is considered an early marker of cardiovascular diseases (8). The prevalence of MAU varies from about 20 to almost 50% in essential hypertension according to the previous studies (9, 10). In general, renal dysfunction and hypertension interact with each other. Moreover, renal abnormality can facilitate the secretion of renin, activating the renin–angiotensin–aldosterone system, which contributes to the development of hypertension. Further, primary or secondary hypertension deteriorates the kidney function and results in renal complications. However, in previous studies, elevated RHR failed to predict MAU in patients with primary hypertension who were treatment naïve or not treated with heart rate-lowering drugs (11, 12). Treatment and blood pressure control are crucial for hypertension management. Therefore, it is important to clarify whether the possible detrimental impact of RHR on MAU is affected by antihypertensive agents or blood pressure status. In this study, we analyzed the data from one of the largest health information sources in the United States, aiming to explore the relationship between RHR and MAU in patients with hypertension and the effect of antihypertensive agents on this relationship according to blood pressure status.

## Methods

### Study Population

We used baseline information from the National Health and Nutrition Examination Survey (NHANES) from 2009 to 2018. The NHANES is a population-based cross-sectional survey conducted by the National Center for Health Statistics, a part of the Centers for Disease Control and Prevention. The program aims to evaluate the health condition of citizens in the United States and uses a stratified, multistage, and cluster sampling design for selecting the target population. Approximately 5,000 participants from all parts of the country are enrolled in this survey each year.

A total of 48,981 participants were assessed from 2009 to 2018. Among them, 41,202 individuals without hypertension; 373 individuals with irregular pulse; 82 individuals aged <20 years; and 1,320 individuals with missing important data such as those on marital status, educational level, poverty income ratio, body mass index (BMI), albumin, and total protein were excluded. Finally, 5,692 individuals met the inclusion criteria and were eligible for this study (Supplementary Figure 1).

### Study Variables

Health examination was performed at Mobile Examination Centers (MECs) or homes if the participants were ≥50 years old or unable to come to the MEC. Radial pulse was measured for 30 s, which could be translated to the RHR by doubling the value. A standard protocol was used by well-trained practitioners for blood pressure measurement. Briefly, at least 5 min were required before measuring the blood pressure of participants. Then, participants were seated quietly, and an appropriate blood pressure cuff was chosen based on their mid-arm circumference. Blood pressure was measured three times, and the average systolic blood pressure (SBP) and diastolic blood pressure (DBP) were calculated.

Participants were diagnosed with hypertension if their average SBP was ≥140 mmHg, their average DBP was ≥90 mmHg, or they were undergoing treatment with antihypertensive agents. Moreover, urine samples were collected for measuring the urinary albumin level, creatinine level, and albumin creatinine ratio (ACR). MAU was defined as an ACR of 30–300 mg/g according to the Kidney Disease: Improving Global Outcomes (13). To evaluate the effect of RHR on MAU according to blood pressure status, the study population was divided into three groups—controlled hypertension (SBP ≤140 mmHg and DBP ≤90 mmHg using at least one type of antihypertensive agent), uncontrolled hypertension (SBP >140 mmHg or DBP >90 mmHg using at least one type of antihypertensive agent), and untreated hypertension (without medical treatment).

In addition, data on demographics, education, marital status, and wealth were collected from the computer-assisted personal interviewing system. Individuals who were married, widowed, or separated were considered married individuals. Questionnaires were designed for identifying lifestyles. Moreover, some serum biochemical indicators, including cholesterol and creatinine, were assessed. The CKD–EPI equation was used for calculating the estimated glomerular filtration rate (eGFR) (14).

### Statistical Analysis

Continuous variables, expressed as means ± standard deviations (SDs) for normally distributed data or as medians (interquartile range), were compared using one-way analysis of variance or the Kruskal–Wallis H test, as appropriate. Categorical variables, expressed as numbers (percentages), were compared using the chi-square test.

The odds ratio (OR) for risk of MAU associated with heart rate was calculated using the logistic regression model. The unadjusted model was first used, followed by multivariate-adjusted model. The candidate confounding factors included established MAU risk factors and variables that were significantly different between heart rate quartiles, including age (continuous), sex (male/female), race (Mexican American/Other Hispanic/Non-Hispanic White/Non-Hispanic Black/Other Race - including Multi-Racial), marital status (married/other), BMI (continuous), poverty income ratio (continuous), current drinking status (yes/no), SBP (continuous), total protein (continuous), eGFR (continuous), total cholesterol (continuous), high-density lipoprotein cholesterol (continuous), diabetes (yes/no), β-blockers (yes/no), and calcium channel blocker (CCB) (yes/no). Forward stepwise selection was performed with entry and exit criteria set at *p* = 0.05 and *p* = 0.10, respectively. Heart rate was included in the models as a continuous variable (per 1 SD) and a categorical variable (the lowest heart rate quartile as the reference), respectively. *P* for trend was tested by treating heart rate quartiles as ordinal variables in the analyses.

To investigate the dose–response relationship between risk of MAU and continuous heart rate, the restricted cubic splines in logistic regression was used with three knots (25th, 50th, and 75th heart rate quartile) and 62 per minute (approximately the first knot) as the reference group.

Subgroups analyses were performed using antihypertensive therapy, including angiotensin-converting enzyme inhibitors (ACEIs)/angiotensin-receptor blockers (ARBs) (yes/no), β-blockers (yes/no), CCB (yes/no), diuretics (yes/no). The effect of the interaction between antihypertensive therapy and heart rate on the risk of MAU was tested by comparing the ORs from different subgroups using Z statistic (15). Participants with eGFR <60 mL/min/1.73 m^2^ were excluded for sensitively analysis.

Finally, mediation analysis was performed using PROCESS in SPSS to evaluated the effect of heart rate (mediator) on the association between β-blockers (independent variable) and MAU (dependent variable) (16). All statistical analyses were performed using R software (version 3.6.2, R Foundation for Statistical Computing) and IBM SPSS Statistics for Windows software, V.23.0. A *P*-value of < 0.05 on the two-sided test was considered statistically significant.

## Results

### Clinical Characteristics of Enrolled Participants

**Figure 1:**
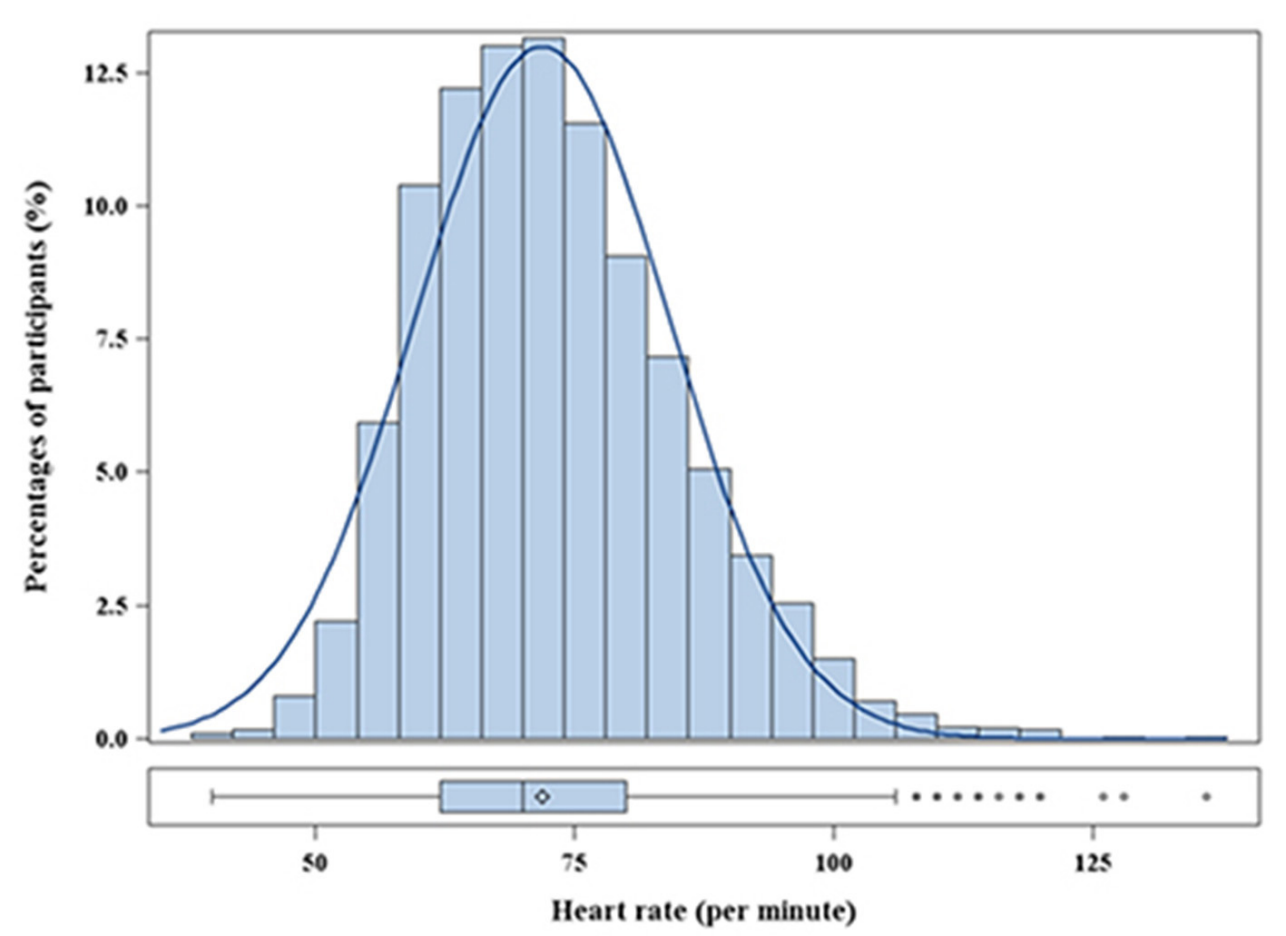
Distribution of heart rate in participants.

In total, 5,692 participants were included in the final analyses. The mean age was 60.8 ± 14.0 years, and 46.1% participants were men. The distribution of heart rate was approximately normal (Figure 1). The mean heart rate was 72 ± 12 bpm. The baseline characteristics, stratified into heart rate quartile (Q1: <62 bpm; Q2: 62–69 bpm; Q3: 70–79 bpm; Q4: ≥80 bpm), are shown in Table 1. Individuals with a higher heart rate tended to be younger and female. Statistically significant trends were noted for all established risk factors, except albumin. In addition, heart rate was inversely associated with the rate of taking β-blockers and CCB. Patients in the Q4 group had a higher BMI and DBP, were more likely to have comorbid diabetes mellitus, and had a lower SBP than those in the Q1 group.

**Table 1:** Characteristics of participants.

	**Total (*n* = 5,692)**	**Q1 (*n* = 1,114)**	**Q2 (*n* = 1,434)**	**Q3 (*n* = 1,682)**	**Q4 (*n* = 1,462)**	***P***
Age, years	61 ± 14	66 ± 12	63 ± 13	60 ± 14	56 ± 15	<0.001
Male, *n* (%)	2,795 (49)	607 (54)	704 (49)	783 (47)	701 (48)	0.037
Heart rate, per minute	70 (62–80)	58 (54–60)	66 (64–68)	74 (72–76)	86 (82–92)	<0.001
Microalbuminuria, %	1,010 (18)	176 (16)	240 (17)	295 (18)	299 (20)	0.002
Systolic BP, mmHg	141.5 ± 19.7	144.3 ± 20.6	142.6 ± 20.6	140.0 ± 19.2	140.1 ± 18.4	<0.001
Diastolic BP, mmHg	74.8 ± 16.1	70.4 ± 14.4	73.8 ± 15.0	75.7 ± 16.6	78.1 ± 16.9	<0.001
Diabetes, *n* (%)	1,234 (22)	212 (19)	295 (21)	372 (22)	355 (24)	0.001
BMI, kg/m^2^	30.8 ± 7.5	29.2 ± 6.1	30.2 ± 7.3	31.1 ± 7.3	32.5 ± 8.5	<0.001
Urine protein, mg/L	26.2 ± 54.9	22.8 ± 44.9	25.1 ± 54.3	25.6 ± 49.9	30.4 ± 66.4	0.001
Albumin, g/L	42 (40–44)	42 (40–44)	42 (40–44)	42 (40–44)	42 (40–44)	0.194
Total protein, g/L	71 (68–75)	70 (67–73)	71 (68–74)	72 (69–75)	72 (69–76)	<0.001
eGFR, ml/min/1.73 m^2^	84.4 (67.8–99.0)	77.3 (63.0–92.5)	83.0 (68.0–96.2)	86.8 (69.1–100.5)	89.4 (72.1–104.7)	<0.001
Total cholesterol, mmol/L	5.02 ± 1.12	4.86 ± 1.11	5.01 ± 1.06	5.04 ± 1.15	5.12 ± 1.13	<0.001
HDL cholesterol, mmol/L	1.37 ± 0.44	1.42 ± 0.43	1.38 ± 0.44	1.37 ± 0.43	1.33 ± 0.44	<0.001
HbA1c, %	5.8 (5.4–6.2)	5.7 (5.4–6.1)	5.8 (5.4–6.2)	5.8 (5.4–6.2)	5.8 (5.4–6.4)	<0.001
Antihypertensive agents, *n* (%)	2,850 (50)	601 (54)	731 (51)	836 (50)	682 (47)	<0.001
ACEI/ARB	1,393 (24)	266 (24)	379 (26)	411 (24)	337 (23)	0.305
β blockers	1,093 (19)	349 (31)	324 (23)	253 (15)	167 (11)	<0.001
Calcium channels blockers	874 (15)	202 (18)	218 (15)	252 (15)	202 (14)	0.024
Dihydropyridine	767 (13)	178 (16)	195 (14)	217 (13)	177 (25)	0.031
Non- Dihydropyridine	107 (15)	24 (2)	23 (3)	35 (2)	25 (2)	0.649
Diuretic agents	1,322 (23)	260 (23)	329 (23)	396 (24)	337 (23)	0.976
Race, *n* (%)						0.126
Mexican American	647 (11)	115 (10)	165 (12)	176 (10)	191 (13)	
Other Hispanic	499 (9)	76 (7)	143 (10)	161 (10)	119 (8)	
Non-hispanic white	2,384 (42)	494 (44)	596 (42)	670 (40)	624 (43)	
Non-hispanic black	1,504 (26)	305 (27)	367 (26)	457 (27)	375 (26)	
Other race - including multi-racial	658 (12)	124 (11)	163 (11)	218 (13)	153 (10)	
Education, *n* (%)						0.098
Less than high school	1,434 (25)	283 (25)	364 (25)	398 (24)	389 (27)	
High school graduate/GED or equivalent	1,408 (25)	260 (23)	333 (23)	434 (26)	381 (26)	
College or AA degree or above	2,850 (50)	571 (51)	737 (51)	850 (51)	692 (47)	
Marital status, *n* (%)						<0.001
Married	3,023 (53)	648 (58)	771 (54)	902 (54)	702 (48)	
Other	2,669 (47)	466 (42)	663 (46)	780 (46)	760 (52)	
PIR, %	2.04 (1.11–3.91)	2.27 (1.23–4.13)	2.11 (1.16–3.97)	2.08 (1.10–4.09)	1.77 (1.00–3.47)	<0.001
Drink, *n* (%)						0.059
<3 cups/day	2,645 (46)	541 (49)	651 (45)	824 (49)	629 (43)	
≥3 cups/day	613 (11)	84 (8)	138 (10)	165 (10)	226 (15)	

*BMI, body mass index; PIR, poverty income ratio; BP, blood pressure; eGFR, estimated glomerular filtration rate; HDL, high-density lipoprotein; ACEI, angiotensin-converting enzyme inhibitors; ARB, angiotensin receptor blockers; HbA1c, glycosylated hemoglobin*.

Table 2 shows the comparison of baseline characteristics between participants who were treated with antihypertensive agents and those who were not. The blood pressure and heart rate burden were heavier in the untreated group than in the treated group. Patients in the treated group tended to be older and were more likely to have diabetes and obesity than those in the untreated group.

**Table 2:** Characteristics of individuals with hypertension according to the usage of antihypertensive drugs.

	**Treated (*n* = 2,808)**	**Untreated (*n* = 2,884)**	***P***
Age, years	63 ± 13	59 ± 15	<0.001
Male, *n* (%)	1,276 (45)	1,519 (53)	<0.001
Heart rate, per minute	70 (62, 78)	72 (64, 80)	<0.001
Microalbuminuria, %	487 (17)	523 (18)	<0.001
Systolic BP, mmHg	131.9 ± 18.9	150.9 ± 15.5	<0.001
Diastolic BP, mmHg	69.7 ± 14.2	79.7 ± 16.3	<0.001
Diabetes, *n* (%)	800 (28)	434 (15)	<0.001
BMI, kg/m^2^	31.6 ± 7.5	30.1 ± 7.4	<0.001
Urine protein, mg/L	9.5 (4.7, 22.9)	10.6 (4.9, 25.1)	0.01
Albumin, g/L	42 (39, 44)	42 (40, 44)	<0.001
Total protein, g/L	71 (68, 74)	72 (69, 75)	<0.001
eGFR, ml/min/1.73 m^2^	80.5 (63.8, 95.1)	88.3 (72.1, 102.6)	<0.001
Total cholesterol, mmol/L	4.83 ± 1.09	5.20 ± 1.11	<0.001
HDL cholesterol, mmol/L	1.34 ± 0.42	1.41 ± 0.45	<0.001
HbA1c, %	5.8 (5.5, 6.4)	5.7 (5.4, 6.1)	<0.001

*BMI, body mass index; BP, blood pressure; eGFR, estimated glomerular filtration rate; HDL, high-density lipoprotein; HbA1c, glycosylated hemoglobin*.

### Association Between Heart Rate and MAU Risk

**Figure 2:**
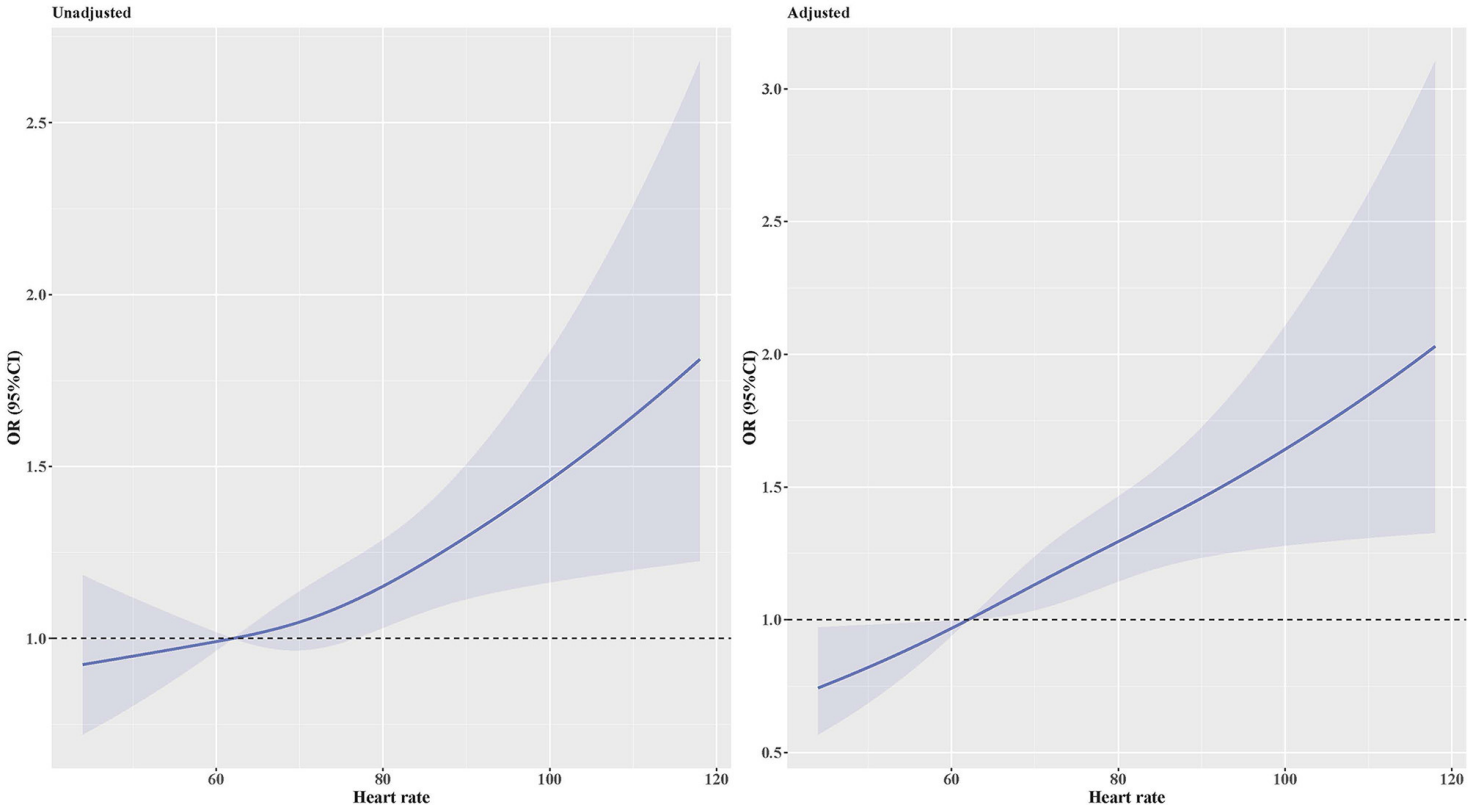
OR and 95% CI for the risk of MAU along with the changes in heart rate according to the restricted cubic splines regression model. Adjusted for body mass index, poverty income ratio, systolic blood pressure, total protein, estimated glomerular filtration rate, diabetes, β-blockers, and calcium channel blockers. *P*-values for non-linear was 0.479 in the unadjusted model and 0.673 in the adjusted model. CI, Confidence interval; OR, Odds ratio.

**Figure 3:**
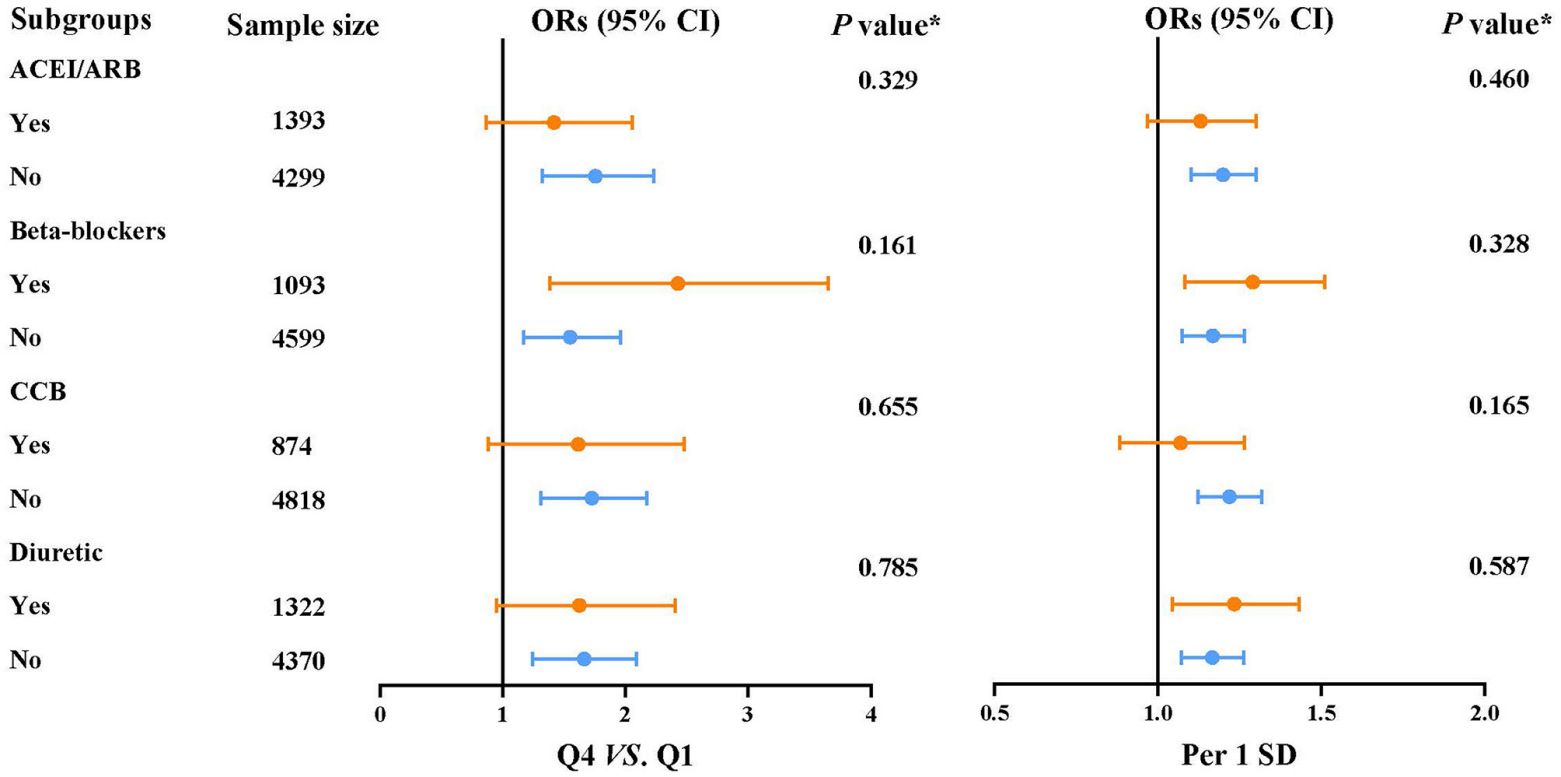
Subgroup analyses for the association between the risk of microalbuminuria and heart rate.

The prevalence of MAU was 18%. After multivariable adjustment (Table 3), per 1 SD increase of heart rate was associated with a 18.4% increase of the risk of MAU (OR: 1.184, 95% confidence interval [CI]: 1.101–1.274, *P* < 0.001). Compared to participants in the first heart rate quartile, those in the third (OR: 1.361, 95% CI: 1.092, 1.695, *P* = 0.006) and fourth (OR: 1.623, 95% CI: 1.295, 2.035, *P* < 0.001) heart rate quartile had a significant higher risk of MAU. The association between heart rate and MAU remained significant among participants with controlled and untreated hypertension, but not in those with uncontrolled hypertension. The dose–response relationship showed that the non-linear relationship between heart rate and MAU was not significant (Figure 2). The relationship between heart rate and the risk of MAU was evaluated among subgroups stratified by antihypertensive agents (Supplementary Table 1). There was no significant interaction effect of antihypertensive agents and RHR on the risk of MAU (Figure 3).

**Table 3:** Association between the risk of microalbuminuria and heart rate.

		**Total ^a^** **(*****n*** **=** **5,692)**		**Controlled ^b^** **(*****n*** **=** **1,937)**		**Uncontrolled ^c^** **(*****n*** **=** **871)**		**Untreated ^d^** **(*****n*** **=** **2884)**	
		**OR (95% CI)**	***P***	**OR (95% CI)**	***P***	**OR (95% CI)**	***P***	**OR (95% CI)**	***P***
Unadjusted	Q1	1		1		1		1	
	Q2	1.071 (0.866, 1.325)	0.525	1.207 (0.787, 1.853)	0.389	0.901 (0.595, 1.365)	0.623	1.233 (0.900, 1.689)	0.191
	Q3	1.134 (0.924, 1.390)	0.229	1.267 (0.839, 1.914)	0.261	0.836 (0.552, 1.266)	0.836	1.405 (1.041, 1.897)	0.026
	Q4	1.370 (1.116, 1.682)	0.003	1.734 (1.146, 2.626)	0.009	1.075 (0.713, 1.622)	0.730	1.519 (1.123, 2.054)	0.007
		*P*_trend_ = <0.002		*P*_trend_ = 0.008		*P*_trend_ = 0.852		*P*_trend_ = <0.004	
	Per 1 SD	1.118 (1.046, 1.195)	0.001	1.198 (1.049, 1.367)	0.008	1.004 (0.871, 1.158)	0.951	1.146 (1.045, 1.257)	0.004
Adjusted	Q1	1		1		1		1	
	Q2	1.170 (0.935, 1.464)	0.170	1.239 (0.798, 1.922)	0.340	0.984 (0.636, 1.522)	0.942	1.233 (0.888, 1.713)	0.211
	Q3	1.361 (1.092, 1.695)	0.006	1.325 (0.865, 2.030)	0.195	0.937 (0.604, 1.455)	0.773	1.578 (1.150, 2.164)	0.005
	Q4	1.623 (1.295, 2.035)	<0.001	1.705 (1.104, 2.632)	0.016	1.325 (0.847, 2.070)	0.217	1.659 (1.200, 2.293)	0.002
		*P*_trend_ <0.001		*P*_trend_ = 0.015		*P*_trend_ = 0.292		*P*_trend_ = 0.001	
	Per 1 SD	1.184 (1.101, 1.274)	<0.001	1.184 (1.030, 1.360)	0.017	1.092 (0.935, 1.275)	0.269	1.197 (1.083, 1.323)	<0.001

a *After forward stepwise selection, the adjusted variables included body mass index, poverty income ratio (PIR), systolic blood pressure (SBP), total protein, estimated glomerular filtration rate (eGFR), diabetes, beta-blockers, and calcium channel blockers*.

b *After forward stepwise selection, the adjusted variables included PIR, SBP, total protein, eGFR, and diabetes*.

c *After forward stepwise selection, the adjusted variables included SBP, eGFR, and diabetes*.

d *After forward stepwise selection, the adjusted variables included BMI, PIR, SBP, total protein, eGFR, and diabetes*.

*CI, confidence interval; OR, odds ratio; SD, standard deviation*.

### Mediation Analysis of Heart Rate on Association Between β-Blockers/Non-DHP and MAU

**Figure 4:**
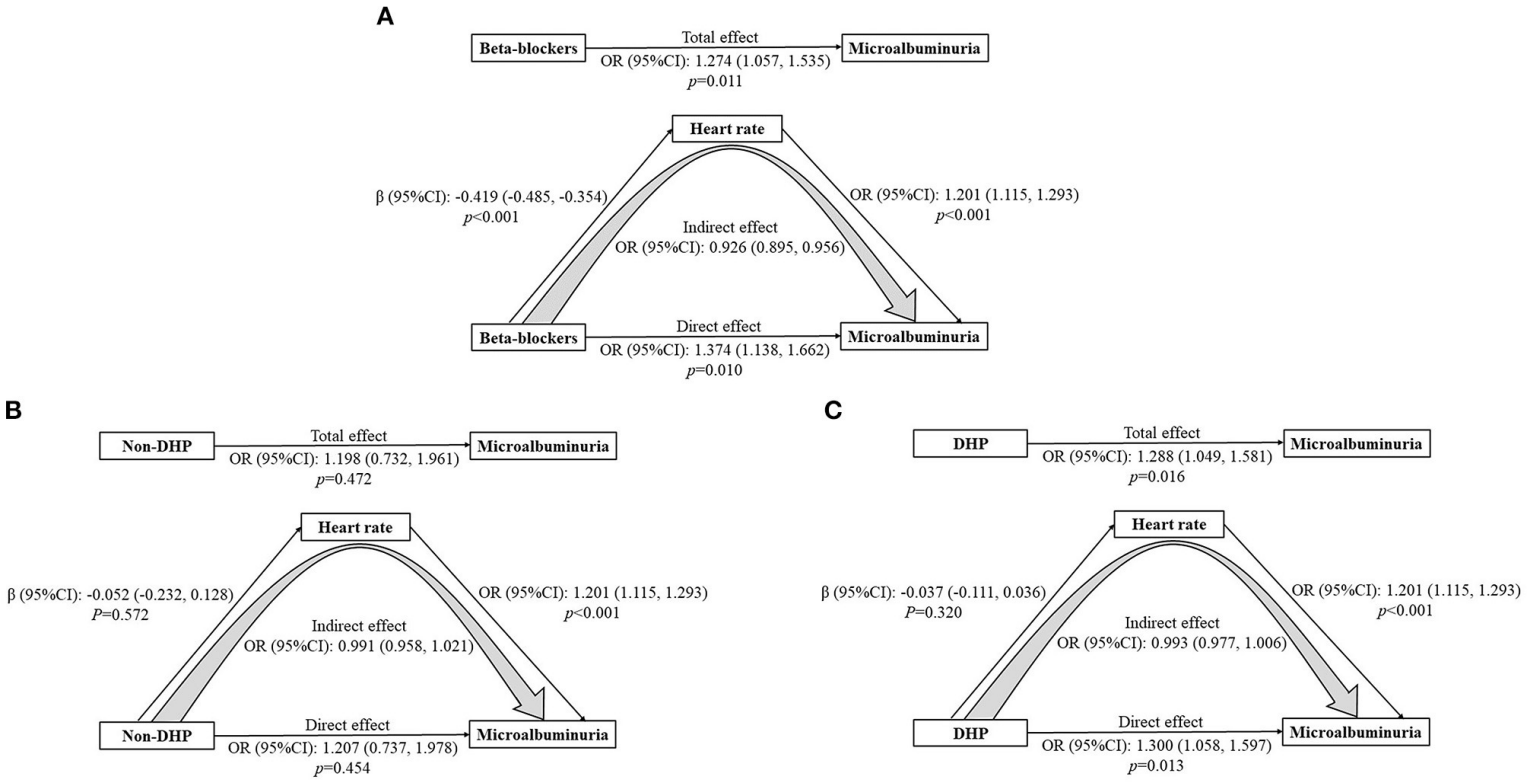
**(A-C)** Mediation analysis of heart rate on the association between β-blockers/non-DHP and microalbuminuria. Adjusted for age, sex, body mass index, poverty income ratio, systolic blood pressure, total protein, estimated glomerular filtration rate, diabetes, and calcium channel blockers. CI, Confidence interval; OR, Odds ratio.

Mediation analysis was further performed to explore the possible effect of heart rate on the association between β-blockers and MAU (Figure 4A). A significant indirect effect on the relationship between β-blockers and MAU prevalence was identified through heart rate (OR: 0.926, 95% CI: 0.895, 0.956). However, there was also a significant direct effect, suggesting that β-blockers were associated with a 37.4% increase in the risk of MAU (OR: 1.374, 95% CI: 1.138, 1.662, *P* = 0.010). Thus, we assessed the total effect of β-blockers on the risk of MAU, which indicated a detrimental role of β-blockers (OR: 1.274, 95% CI: 1.057, 1.535, *P* = 0.011).

Besides, mediation analysis was performed to evaluate the potential effect of heart rate on the relationship between non-dihydropyridine (non-DHP) and MAU as well (Figure 4B). The association between non-DHP and MAU was not significant (OR 1.207, 95% CI: 0.737, 1.978, *P* = 0.454). On the contrary, DHP was related to an increased prevalence of MAU (OR: 1.300, 95% CI: 1.058, 1.597, *P* = 0.013) (Figure 4C).

## Discussion

In the present study, the relationship between elevated RHR and increased risk of MAU in patients with hypertension was significant. Except in uncontrolled hypertension, the detrimental effect of RHR on the incidence of MAU was prominent in different blood pressure statuses, which was minimally affected by antihypertensive agents. Among the four types of antihypertensive agents, β-blockers could indirectly reduce the risk of MAU due to its negative chronotropic effect, while the total effect of β-blockers on the prevalence of MAU was detrimental. Similarly, dihydropyridine calcium channel blockers were associated with a higher prevalence of microalbuminuria, but the association between non-dihydropyridine calcium channel blockers and microalbuminuria was not significant.

In the Framingham study, elevated RHR strongly predicted a high risk of all-cause and cardiovascular mortality in both men and women with hypertension (17). The overactive sympathetic nervous system contributes to the increase in RHR, further promoting atherogenesis in hypertension, thereby deteriorating the cardiovascular prognosis (18, 19). The data from I-SEARCH indicated a high risk of MAU in patients with hypertension and elevated RHR. However, this effect was questioned by other investigators.

Cuspidi et al. pointed out the prevalence of MAU was not affected by the increase in office or 48-h heart rate (11). Similarly, another study reported that there was no correlation between ambulatory heart rate and MAU (12). These results were in contrast to those reported by Böhm's study and our study, possibly because of the following reasons. (a) A specific study population was employed. All participants were from the same race such as Caucasian, and it is unclear whether the prognostic value of heart rate is affected by different race. Moreover, antihypertensive treatment cannot be easily ignored when studying the hypertensive population. In particular, β-blockers, heart rate-lowering drugs, exert a potent effect on the sympathetic nerve and heart rate. (b) Limited number of participants were included. Given the high prevalence of MAU in essential hypertension, inclusion of hundreds of participants may be insufficient.

From our perspective, the prevalence of MAU in participants with uncontrolled hypertension was not affected by elevated RHR, possibly due to the change in the relative proportion of MAU and macroalbuminuria. In the uncontrolled group, the relative proportion of macroalbuminuria increased and the elevated RHR was further associated with a high risk of macroalbuminuria (data not shown). Furthermore, the small sample size of uncontrolled group (*N* = 916) should be taken considered.

Dysfunction of sympathetic nervous system is considered the contributor of increased RHR and blood pressure. However, regional sympathetic nerve activation is different in primary hypertension where renal sympathetic outflow seems to be inactive (18). The cause of hypertensive renal damage may need other explanations. In patients with a cardiovascular risk, the negative influence of elevated RHR on endothelial function was found from a clinical perspective (20). Moreover, endothelial dysfunction aggravated the kidney diseases and MAU is an early-stage biomarker for endothelial dysfunction in a previous study (21). Thus, the high prevalence of MAU observed in our study may result from the endothelial damage caused by elevated RHR, which needs to be further studied.

Bangalore et al. previously conducted a meta-analysis and found that β-blocker-induced decrease of RHR significantly enhanced the risk for developing cardiovascular events (22). It is paradoxical that elevated RHR is closely associated with poor prognosis, while improving the aberrant heart rate by β-blockers further exacerbates cardiovascular risk. Further, regular physical activity attenuated the risk raised by elevated RHR in a previous study (23).

In our study, individuals benefited from the heart rate-lowering treatment because of the linear relationship between MAU and RHR. However, β-blockers may indirectly reduce the high incidence of MAU by decreasing the RHR, although the direct effect of β blockers treatment was detrimental. Interestingly, another heart rate-lowering agents, non-DHP calcium channel blockers, had minimal effect on the association between RHR and MAU. Although the insignificant effect of non-DHP may be explained by the limited users of non-DHP (*n* = 107), it still indicated a harmful role of β blockers in the MAU development. It was found that DHP calcium channel blockers increased the risk of MAU. Given that DHP calcium channel blockers can contribute to the elevation of RHR, it may make sense why MAU became more prevalent.

A similar trend was reported by Böhm et al. (7) they noted a reduction in MAU prevalence in the participants with regular physical activity. Therefore, heart rate reduction is essential for preventing the development of MAU in hypertension, while the use of β-blockers may not suitable due to its potential harm to the kidney.

## Conclusion

Elevated RHR strongly associated with the prevalence of MAU in the patients with controlled and untreated hypertension. A linear relation between RHR and MAU was found in hypertension. However, it might be harmful to use β-blockers for ameliorating the glomerulus damage caused by increased RHR. For the physicians, it may be necessary to regularly detect the albuminuria level and renal function in those who were treated with β-blockers due to hypertension and elevated RHR. For investigators, further studies are needed to determine the causal relationship of β-blockers treatment on renal dysfunction in patients with hypertension. Prospective studies are warranted to comprehensively elucidate the prognostic value of RHR for kidney diseases in hypertension and possible solution for preventing this clinical situation.

### Limitations

This study has several limitations. First, we conducted this study using a cross-sectional database. Thus, a causal relationship cannot be fully studied. Second, some important factors such as smoking status and physical activity were not adjusted in this study. Third, blood pressure was measured on the same day instead of alternate days; therefore, we cannot exclude the white coat effect, and data on 24-h ambulatory heart rate was not collected. However, the RHR was not inferior to the average ambulatory heart rate reported by a previous study (24).

## Data Availability Statement

The datasets presented in this study can be found in online repositories. The names of the repository/repositories and accession number(s) can be found below: https://www.cdc.gov/nchs/nhanes/about_nhanes.htm.

## Ethics Statement

The studies involving human participants were reviewed and approved by https://www.cdc.gov/nchs/nhanes/about_nhanes.htm. The patients/participants provided their written informed consent to participate in this study.

## Author Contributions

XW and YL collected the data for analysis. XP evaluated the statistical significance of related data and wrote this manuscript. YR contributed to the manuscript revision. NL designed the study and interpreted results. All authors read and approved the final manuscript.

## Funding

This work was supported by the National Science Foundation of China (Grant Nos. 81870244 and 81770318) and the Natural Science Foundation of Beijing (Grant No. 7192051).

## Conflict of Interest

The authors declare that the research was conducted in the absence of any commercial or financial relationships that could be construed as a potential conflict of interest.

## Publisher's Note

All claims expressed in this article are solely those of the authors and do not necessarily represent those of their affiliated organizations, or those of the publisher, the editors and the reviewers. Any product that may be evaluated in this article, or claim that may be made by its manufacturer, is not guaranteed or endorsed by the publisher.
