# Supplementary material for: Relationship Between Resting Heart Rate and Microalbuminuria in Adults With Hypertension: National Health and Nutrition Examination Survey 2009–2018
Source: Front Cardiovasc Med. 2022 Apr 12;9:739113. doi: 10.3389/fcvm.2022.739113 (PMC9039360; doi:10.3389/fcvm.2022.739113)
Supplement: Supplementary file 1 [file Data_Sheet_1.DOCX]

Supplementary materials


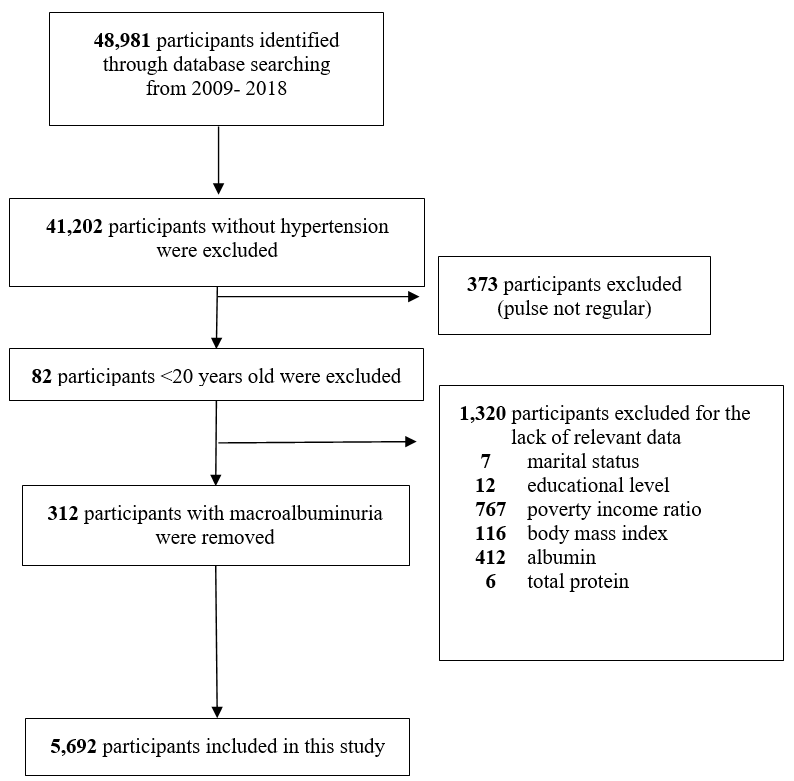


eFigure 1. Study population selection

eTable 1. Subgroup analyses for the association between the risk of microalbuminuria and heart rate.

|  |  | Without | | With | |
| --- | --- | --- | --- | --- | --- |
|  |  | OR (95%CI) | *P* | OR (95%CI) | *P* |
| ACEI/ARB | Q1 | 1 | | 1 | |
|  | Q2 | 1.266 (0.973, 1.646) | 0.079 | 0.893 (0.580, 1.375) | 0.608 |
|  | Q3 | 1.523 (1.180, 1.966) | 0.001 | 0.923 (0.602, 1.416) | 0.715 |
|  | Q4 | 1.715 (1.318, 2.230) | 0.000 | 1.332 (0.865, 2.053) | 0.193 |
|  | P 1 SD | 1.197 (1.102, 1.301) | 0.000 | 1.123 (0.969, 1.302) | 0.125 |
| Beta-blockers | Q1 | 1 | | 1 | |
|  | Q2 | 1.127 (0.864, 1.469) | 0.378 | 1.353 (0.887, 2.063) | 0.160 |
|  | Q3 | 1.302 (1.010, 1.679) | 0.042 | 1.599 (1.018, 2.512) | 0.042 |
|  | Q4 | 1.514 (1.169, 1.960) | 0.002 | 2.246 (1.380, 3.654) | 0.001 |
|  | P 1 SD | 1.166 (1.075, 1.265) | 0.000 | 1.279 (1.083, 1.511) | 0.004 |
| CCB | Q1 | 1 | | 1 | |
|  | Q2 | 1.182 (0.921, 1.516) | 0.188 | 1.097 (0.653, 1.842) | 0.728 |
|  | Q3 | 1.456 (1.142, 1.856) | 0.002 | 0.955 (0.570, 1.599) | 0.860 |
|  | Q4 | 1.706 (1.329, 2.189) | 0.000 | 1.293 (0.762, 2.195) | 0.341 |
|  | P 1 SD | 1.217 (1.124, 1.317) | 0.000 | 1.030 (0.860, 1.234) | 0.749 |
| Diuretic | Q1 | 1 | | 1 | |
|  | Q2 | 1.249 (0.969, 1.611) | 0.086 | 0.895 (0.555, 1.442) | 0.649 |
|  | Q3 | 1.457 (1.136, 1.868) | 0.003 | 1.004 (0.634, 1.590) | 0.986 |
|  | Q4 | 1.628 (1.260, 2.103) | 0.000 | 1.512 (0.950, 2.406) | 0.081 |
|  | P 1 SD | 1.165 (1.074, 1.264) | 0.000 | 1.224 (1.046, 1.433) | 0.012 |
